# Visual saliency influences ethical blind spots and (dis)honesty

**DOI:** 10.3758/s13423-019-01638-1

**Published:** 2019-07-06

**Authors:** Andrea Pittarello, Marcella Frătescu, Sebastiaan Mathôt

**Affiliations:** 1 Department of Psychology, Brooklyn College, City University of New York, 2900 Bedford Avenue, 11210 Brooklyn, New York, NY USA; 2 Department of Psychology, University of Groningen, Groningen, The Netherlands

**Keywords:** Morality, Attention, Eye movements, Blind spots, Pre-registration

## Abstract

Recent work suggests that dishonesty results from ethical blind spots: people’s lack of attention to ethical information. In two experiments (one pre-registered) we used eye tracking to investigate when ethical blind spots emerge, and whether they can be reduced through a simple, non-invasive intervention. Participants reported a Target Digit indicated by a jittery cue that was slightly biased in the direction of another digit (the Second-Cued Digit), which could be either higher or lower than the Target Digit. Participants were paid more for reporting higher digits, and were not penalized for making mistakes, thus providing an incentive to cheat. Results showed that participants frequently made self-serving (and rarely self-hurting) mistakes by reporting the Second-Cued Digit when it was more valuable than the target. Importantly, they rapidly gazed at the digit that they would later report, regardless of whether this report was correct or a self-serving mistake. Finally, we were able to reduce or increase the number of self-serving mistakes by respectively increasing or reducing the visual saliency of the Target Digit. We suggest that increasing the visual saliency of morally desirable options is a promising cost-effective tool to curb dishonesty.

**Electronic supplementary material:**

The online version of this article (10.3758/s13423-019-01638-1) contains supplementary material, which is available to authorized users.

## Introduction

Although people think of themselves as honest – more honest than others even (Bazerman & Tenbrunsel, 2011) – most people occasionally cheat. According to self-concept maintenance theory (Mazar, Amir, & Ariely, 2008), when people are faced with a choice between honesty and dishonesty, they cheat to the extent to which they can justify their misbehavior (Shalvi, Dana, Handgraaf, & De Dreu, 2011). According to bounded ethicality theory (Chugh, Bazerman, & Banaji, 2005), dishonesty results from “ethical blind spots.” Following Bazerman and Tenbrunsel (2011), we define ethical blind spots as situations in which people pay little (or no) attention to ethical considerations when doing so is against their self-interest (for a similar definition, see Bazerman, 2014; Pittarello, Leib, Gordon-Hecker, & Shalvi, 2015). For instance, imagine a CEO conflicted between recalling a malfunctioning product that can hurt people (and thus losing money) or ignoring this issue and leaving the product on the market (and thus earning money). In this situation, the financial incentive to keep the product on the market can avoid the CEO from paying attention to the ethical issue, in turn increasing dishonesty. At times, blind spots are unintentional (e.g., when people are unaware of a conflict of interest), while at other times they represent an intentional justification strategy, such as when people avoid attending to information that will prevent them from obtaining undeserved gains (see Jacobsen, Fosgaard, & Pascual-Ezama, 2018; Pittarello, Motro, Rubaltelli, & Pluchino, 2016).

Recent work has used eye tracking to show that blind spots emerge more often in tempting and ambiguous situations, where ambiguity serves as a justification to do wrong; this is in line with the notion of self-concept maintenance (i.e., it allows people to maintain a positive self-image) as well as bounded ethicality. For instance, Pittarello et al. (2015) asked participants to report the outcome of a die roll (one of six dice) appearing closest to a fixation cross to determine their pay, with higher rolls corresponding to higher payoffs. Ambiguity was manipulated by displaying the fixation cross on the midpoint of either the right or left side of the die. At times, the die second closest to the fixation cross was higher (and thus more profitable) than the target die. In these ambiguous settings, participants exhibited blind spots: They looked more at the higher die, even if it was not the target.

Our research question is to understand when, during the decision process, blind spots arise. We hypothesize that they occur rapidly, and base our prediction on research on behavioral ethics that employs a dual-process perspective (Evans, 2008; Haidt, 2007; Kahneman, 2011; Strack & Deutsch, 2004); according to research, in tempting situations, people’s first reaction is to serve their self-interest. As a case in point, Shalvi et al. (2012, 2013) found that people cheated more under time pressure than when given ample time. Similarly, Mead et al. (2009) and Gino et al. (2011) found that people cheat more when they lack self-control and when their cognitive capacities are reduced. Finally, work on neuroscience showed that areas of the brains associated with self-control are activated when people avoid lying (Greene & Paxton, 2009). To sum, this work shows that in tempting situations, people’s initial motivation is to lie.

While motivation affects cheating, in tempting situations it also makes people perceive and process information in a self-serving way (Balcetis & Dunning, 2006). This suggests that when cheating pays off and justifications are available, people should quickly gaze at tempting (yet dishonest) information that would allow them to earn undeserved money while in turn paying little attention to honest (but less profitable information). Thus, we predict that when facing the opportunity to cheat, blind spots manifest quickly during the decision process.

Eye tracking is a powerful tool to detect when ethical blind spots arise, because fast eye movements are largely beyond voluntary control, and reflect involuntary processes such as previous rewards (we tend to first look at things that were previously rewarded, regardless of whether they will be rewarded again) and visual saliency (we tend to first look at things that have sharp edges, high luminance contrast, etc.) (Awh, Belopolsky, & Theeuwes, 2012; Theeuwes, Kramer, Hahn, & Irwin, 1998).

Hochman and colleagues (2016) tested whether people immediately look at profitable, yet “dishonest,” options by asking participants to report whether there were more dots on either the left or right side of a square. In the experimental trials, participants received more money if they reported one side of the square (e.g., always the left side) irrespective of the accuracy of the response. This provided them with an opportunity to cheat. By tracking eye movements, the authors found that when participants cheated, they tended to look immediately at the highly rewarded half, rather than the half that actually contained more dots. This finding is important and provides initial support for the rapid emergence of blind spots. However, in this experiment, the rewarded side was kept constant throughout blocks of many trials; therefore, the results cannot tell us whether the eye movements toward the highly rewarded side indeed reflected a fast tendency to cheat, or rather a strategy that built up over time.

Overall, these findings show that profitable (but morally undesirable) information attracts attention at the expense of less profitable (but morally desirable) information, thus shaping ethical blind spots. Therefore, interventions that redirect people’s attention back toward morally desirable information should reduce ethical blind spots and promote honesty. This is in line with the REVISE framework put forth by Ayal et al. (2015). According to this model, providing subtle cues that increase the saliency of ethical criteria can decrease dishonesty in tempting and ambiguous situations. To be effective, these cues should be timely, which means that they should be presented right before people have the opportunity to cheat. Building on this framework, we reasoned that one possible intervention involves *visual* saliency: The extent to which visual stimuli stand out from the environment, for example because they are high in contrast or contain sharp edges (Itti, Koch, & Niebur, 1998). Salient stimuli capture people’s attention (Yantis & Jonides, 1984), and in real-life settings tend to be preferred over less salient stimuli (Orquin, Scholderer, & Jeppesen, 2012).

**Fig. 1:**
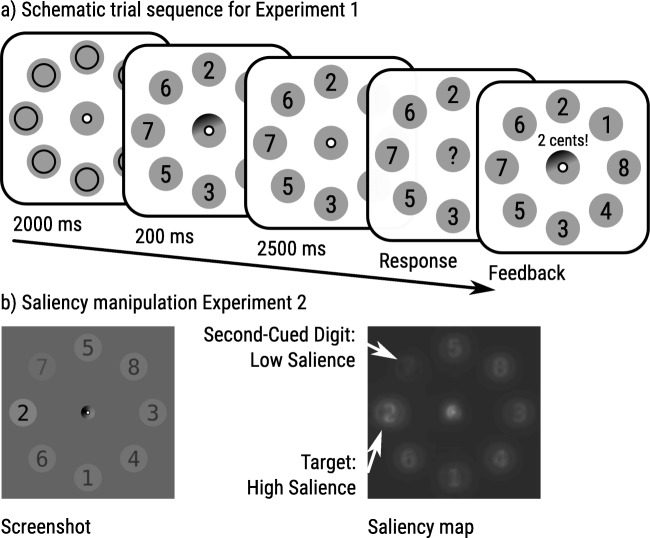
(**a**) A schematic representation of the experimental procedure for Experiments 1 and 2. In this example, the cue points halfway between the “2” and the “6,” which we refer to as 50% ambiguity. (**b**) In Experiment 2, the visual saliency of the Target Digit was either increased or decreased relative to that of the Second-Cued digit. In this example, the saliency of the Target Digit (“2”) is increased relative to the saliency of the Second-Cued Digit (“7”). The saliency map was generated with the Python Saliency Map library (https://github.com/mayoYamasaki/saliency-map)

In two experiments (one pre-registered) we asked participants to report a Target Digit (among multiple digits) that was indicated by a Cue (Fig. 1). The cue was a fuzzy and jittery line-segment that was slightly biased towards another digit, which we call the Second-Cued Digit. Participants were either paid for accurately reporting the Target Digit (Accuracy condition) or were paid based on the value of the reported digit, regardless of accuracy, such that reporting a higher digit yielded a higher payoff (Report condition). On some trials, the Second-Cued Digit was higher than the Target Digit. Here, participants in the Report condition could make self-serving (but not self-hurting) mistakes to increase their payoff. In other words, dishonesty would manifest itself as a tendency to report the Target Digit less often (and the Second-Cued Digit more often) when it is lower than the Second-Cued Digit, compared to when it is higher than the Second-Cued Digit.

Experiment 1 tested whether, when making self-serving mistakes, participants’ initial eye movements are directed toward the tempting digit even when this is the dishonest option; if so, this would suggest that participants’ reports are caused by the rapid emergence of blind spots.

Experiment 2 tested whether increasing the visual saliency of the Target Digit would reduce self-serving mistakes. Crucially, the digit stimuli were large enough to be discriminated in peripheral vision; therefore, participants could directly look at the digit that attracted attention most, without first needing to scan the display with a series of eye movements.

## Methods

### General procedure and apparatus

Participants were recruited through the student subject pool of the University of Groningen in exchange for course credit and an amount of money based on their performance. On average, participants earned €6 (~US$7.07). The study was approved by the local ethics committee of the University of Groningen, and all participants read and signed a consent form before participation. In both experiments, participants sat approximately 60 cm away from a 21-in. screen (resolution 1,920 × 1,080 px). Eye movements were recorded with an Eye Tribe eye tracker (Denmark; sampling rate = 30 Hz, accuracy = 0.50°) and stimuli were presented using OpenSesame 3.1 (Mathôt, Schreij, & Theeuwes, 2012). Following a nine-point calibration, participants received verbal and written instructions. Both experiments lasted ±35 mins, after which participants were thanked and debriefed. All data and experimental materials can be found at https://osf.io/gc3sz/?view_only=c416fb3279084930897d44ecb6558a66.

## Experiment 1

### Methods

#### Participants

Sixty participants (83% female, M_age_= 21.44 years) took part in the study. Sample size was determined based on previous work (Pittarello et al., 2015), although we used 50% more participants to ensure a high statistical power. All participants had normal or corrected-to-normal vision. Fourteen additional participants completed the experiment but did not pass the manipulation check at the end of the experiment. These participants were replaced and excluded from the analysis.

#### Procedure

Participants saw a circular arrangement of eight randomly presented digits (from 1 to 8), one of which (the Target Digit) was indicated by a central jittery cue. After 2,500 ms, a central question mark appeared, and participants indicated the Target Digit (Fig. 1a). Participants in the Accuracy condition (*N*=30) earned €0.06 (~$0.07) for accurately reporting the Target Digit, and €0 otherwise. Participants in the Report condition (*N*=30) were paid according to the digit they reported, with higher digits meaning higher payoffs (i.e., 1 = €0.01, 2 = €0.02, 3 = €0.03, 4 = €0.04, 5 = €0.05, 6 = €0.06, 7 = €0.07, and 8 = €0.08). Within blocks, we varied the ambiguity of the cue, such that it pointed slightly (10%) or moderately (30%) in the direction of the Second-Cued Digit, or exactly in-between the Target Digit and the Second-Cued Digit (i.e., fully ambiguous; 50%). On some trials, the Second-Cued Digit was higher (i.e., more profitable) than the Target Digit (tempting trials), while on other trials it was lower (i.e., less profitable) than the Target Digit (non-tempting trials).

The complete experimental design included one between-subject factor (condition: Accuracy vs. Report) and two within-subject factors (Cue ambiguity: low vs. medium vs. high; and Second-Cued Digit value [temptation]: higher vs. lower than the cued digit). Participants performed six practice trials followed by five blocks of 24 trials and received feedback about their cumulative earnings after each trial. At the end of the experiment, participants were presented with the following manipulation check: “Please pay attention! We want to make sure that you understood how you earned money in this task. You received: ..”. Participants could choose one of the following options: “More money for a correct than an incorrect answer (regardless of whether you reported a high or low digit)” or “More money for reporting a high than a low digit (regardless of whether your answer was correct).” The exact phrasing of the manipulation check was changed halfway the experiment, when it became clear that participants frequently failed the manipulation check because they did not understand the question (while their responses suggested that they had understood the task). Participants also filled out a personality questionnaire either before or after the experiment. These data are not reported here since they were outside the scope of our investigation. However, including these measures did not change any of results reported below.

### Results

#### Behavior

To test for self-serving mistakes, we conducted a general linear mixed-effects model (GLM) with proportion of target-digit reports (binomial) as dependent variable, Condition, Second-Cued Value, and Cue ambiguity as fixed effects. We also added two interactions that were of clear theoretical interest, because these reflect possible different patterns of behavior between the report and accuracy conditions: Cue ambiguity × Condition, and Second-Cued Value × Condition. Our models included by-participant random intercepts for all fixed effects, but no random slopes (including random slopes often caused failures to converge). Fixed effects were centered, such that their mean was 0, with steps of 1 between consecutive levels. Results showed a main effect of Condition, with participants reporting the Target Digit on 73% of the trials in the Accuracy condition versus 47% of the trials in the Report condition, B=-1.58, SE=.27, Z=-5.87, *p* <.0001. We found a main effect of Cue ambiguity, B=-.98, SE=.07, Z=-14.13, *p* <.0001 showing that the Target Digit was reported less frequently when the location of the cue was more ambiguous. These main effects were qualified by a significant Condition × Cue ambiguity interaction, B=.69, SE=.14, Z=4.97, *p* <.0001, indicating that participants were less sensitive to the location of the cue in the Report condition than in the Accuracy condition, where greater ambiguity corresponded to a higher rate of mistakes. There was a main effect of Second-Cued Value, B=-.96, SE=.13, Z=-7.21, *p* <.0001, indicating that accuracy was lower if the Second-Cued Digit was higher than the Target Digit (53%) versus lower than the Target Digit (67%). Importantly, there was a Condition × Second-Cued Value interaction, B=-1.78, SE=.27, Z=-6.61, *p* <.0001. Participants in the Accuracy condition reported the Target Digit equally often regardless of whether it was lower (72%) or higher (73%) than the Second-Cued Digit. However, those in the Report condition made self-serving mistakes: They reported the Target Digit less often when it was lower (34%) versus higher (60%) than the Second-Cued Digit, conceptually replicating prior work by Pittarello et al. (2015).

**Fig. 2:**
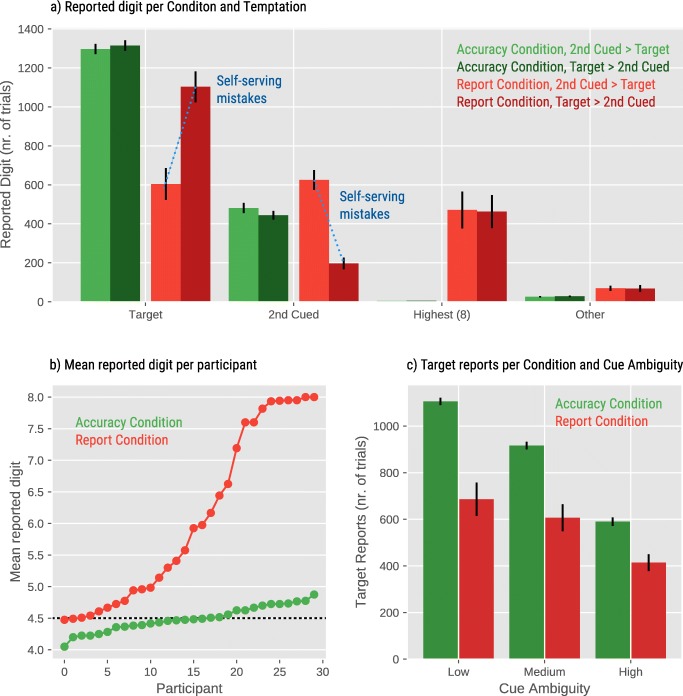
Behavioral results for Experiment 1. On the Y-axis we report the total number of trials (for panel **a** and **c**) on which participants reported their choice. Overall, there were 7,200 trials in Experiment 1 (3,600 trials per condition). Error bars represent standard errors

The distribution of mean reported digits for individual participants in the Report condition is mostly continuous, even though we observed a cluster of participants who consistently reported “8” (Fig. 2b). We label the latter group as “brazen liars” (see Weisel & Shalvi, 2015) who seemed not to be affected by our manipulations. For robustness purposes, we conducted the same analyses after removing participants whose average reports were 7.5 or higher. Doing so did not meaningfully change the results reported here (see Supplementary Material, Fig. 1S).

Overall, the behavior showed that if participants were paid based on the digit that they reported, (a) they were less sensitive to the cue, (b) they frequently misreported the Second-Cued Digit when it was more valuable than the target (self-serving mistakes), (c) they frequently misreported the highest digit, and (d) the tendency to cheat was distributed more or less continuously in the participant sample, meaning that cheaters exist in all sizes: some cheat very little, some a bit more, and others consistently reported “8.”

#### Eye movements

We first created seven temporal bins of equal duration. Next, for each bin separately, and for each condition (Accuracy vs. Report) we conducted two separate GLMs: The first included the proportion of fixations on the Target Digit (binomial) as the dependent variable, the main effects of Target Reported (whether or not the target was reported on a given trial) and Second-Cued Value, and their interaction as fixed effects. The second was identical but included the proportion of fixations on the Second-Cued Digit as the dependent variable. As for the behavioral analysis, models included by-participant random intercepts for all fixed effects, but no random slopes. Fixed effects were centered, such that their mean was 0, with steps of 1 between consecutive levels.

**Fig. 3:**
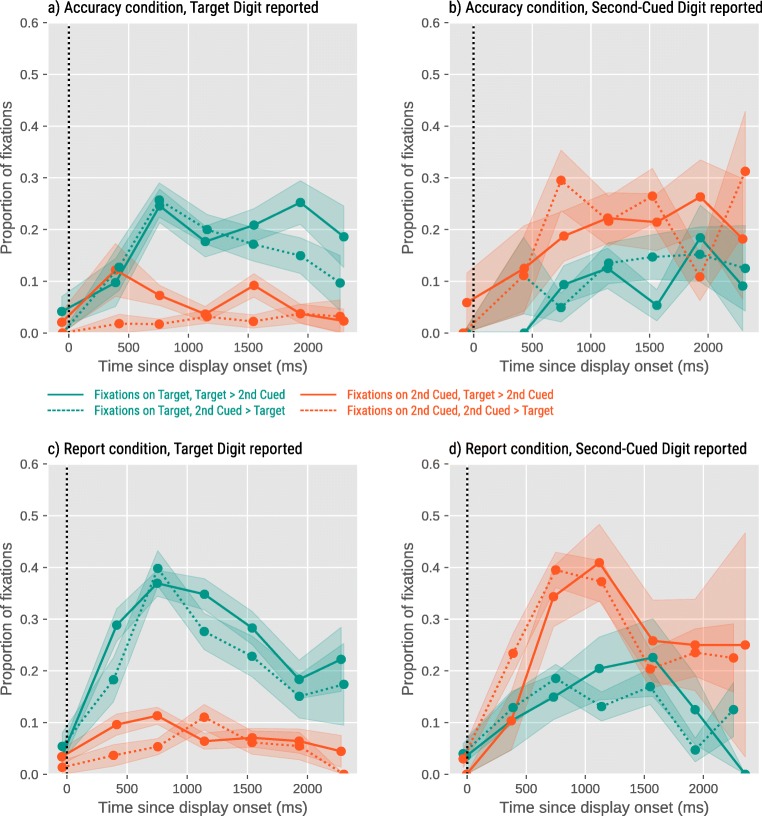
Eye-movement results for Experiment 1 split by condition (**a, b**: Accuracy; **c, d**: Report) and the digit that was reported by the participant (**a, c**: Target; **b, d**: Second-Cued Digit). In all cases, participants look mostly at the digit that they will report later. The data shown in (**b**) are somewhat noisy, because there were relatively few trials in the Accuracy condition on which participants reported the Second-Cued Digit. Error bars represent standard errors

Figure 3 shows the proportion of the eye fixations on the Target Digit (teal line) and Second-Cued Digit (orange line) for the Accuracy and Report condition over time, where time was divided into seven bins. In the Report condition, there was a main effect of Target Reported for bin 2, B=1.26, SE=.28, Z=4.36, *p* <.0001, bin 3, B=1.35, SE=.17, Z=7.78, *p* <.0001, bin 4, B=1.40, SE=.21, Z=6.60, *p* <.0001, bin 5, B=.82, SE=.23, Z=3.52, *p* <.001, bin 6, B=1.37, SE=.38, Z=3.63, *p* <.001, and marginally for bin 7, B=.92, SE=.49, Z=1.88, *p* =.06 on the proportion of fixations on the Target Digit. Similarly, there was a main effect of Second-Cued Reported for bin 2, B=1.22, SE=.41, Z=2.99, *p* <.0001, bin 3, B=2.03, SE=.20, Z=9.97, *p* <.0001, bin 4, B=2.28, SE=.25, Z=9.21, *p* <.0001, bin 5, B=1.78, SE=.32, Z=5.55, *p* <.0001, and bin 6, B=2.05, SE=.39, Z=5.24, *p* <.0001 on the proportion of fixations on the Second-Cued Digit. There was no main effect of temptation above and beyond the effect of the reported digit (no *p* < .05).

The same analyses in the Accuracy condition showed that there was a main effect of the Target Reported for bin 3, B=1.94, SE=.42, Z=4.67, *p* <.0001, bin 4, B=.63, SE=.01, Z=377.98, *p* <.0001, and bin 5, B=.91, SE=.38, Z=2.41, *p* =.015. There was an effect of Second-Cued Digit Reported for bin 3, B=2.49, SE=.44, Z=5.60, *p* <.0001, bin 4 B=2.23, SE=.37, Z=5.93, *p* <.0001, bin 5 B=1.93, SE=.40, Z=4.86, *p* <.0001, bin 6 B=1.72, SE=.49, Z=3.54, *p* <.001, and bin 7 B=2.47, SE=.86, Z=2.87, *p* <.01. Additionally, we found an interaction between Second-Cued Digit Reported and Second-Cued Value for bin 3, B=2.03, SE=.83, Z=2.45, *p* =.01, and for bin 5, B=1.77, SE=.79, Z=2.24, *p* =.03, indicating that the effect of Second-Cued Digit reported was stronger when the Second-Cued Digit was higher than the Target Digit (see Fig. 2S in the Supplementary Material for the pattern without “brazen liars”).

In the report condition, where cheating was possible, the finding that participants quickly look at higher digits – even when, in the report condition, these digits were the dishonest options – suggests that ethical blind spots (and subsequent self-serving mistakes) arise quickly as soon the digits appear.

## Experiment 2

Experiment 2 tested whether it is possible to limit ethical blind spots and reduce self-serving mistakes through a counteracting bias that guides participants away from the Second-Cued Digit. To accomplish this, we made the Second-Cued Digit either less salient than the Target Digit, with the aim to reduce cheating, or more salient, with the aim to increase cheating. Experiment 2 included only the Report condition and was preregistered as https://osf.io/n7xu6/.

### Methods

#### Participants

Thirty participants (72.7% female, M_age_= 24.18 years) took part in the study (i.e., the same sample size as in each condition of Experiment 1). All participants had normal or corrected-to-normal vision. Four additional participants completed the experiment but did not pass the manipulation check at the end of the experiment (see *Procedure* of Experiment 1). These participants were replaced and excluded from the analysis.

*Procedure*

The procedure was identical to the Report condition of Experiment 1 with the following exceptions. First, the ambiguity of the cue was fixed at an intermediate level (30%). Second, the Second-Cued Digit was either more or less salient than the Target Digit. The saliency of all other digits was intermediate. We manipulated the saliency of the stimuli by reducing their contrast with the background, such that high saliency corresponded to a contrast of 100% (as in Experiment 1), intermediate saliency to a contrast of 50%, and low saliency to a contrast of 25%. The complete experimental design included two within-subject factors (value of the Second-Cued Digit: higher [tempting] vs. lower [non-tempting] than the Target Digit; and saliency of the Second-Cued Digit: more vs. less salient than the Target Digit). Participants performed eight practice trials, followed by eight blocks of 28 trials.

#### Behavior

**Fig. 4:**
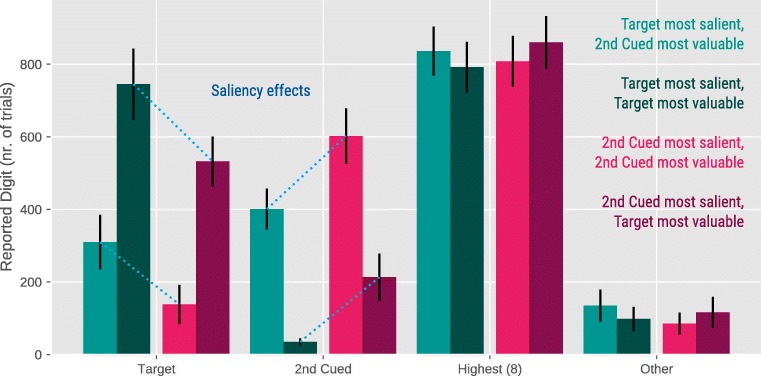
Behavioral results for Experiment 2. On the Y-axis we report the total number of trials on which participants reported their choice. Overall, there were 6,720 trials in Experiment 2. Error bars represent standard errors

Similar to Experiment 1, we conducted a GLM with proportion of target-digit reports as dependent variable, and Temptation, Saliency, and the Temptation × Saliency interaction as fixed effects. The pattern of self-serving mistakes replicates that observed in Experiment 1. We found a main effect of Temptation, B=-4.12, SE=.64, Z=-6.37, *p* <.0001, showing that participants reported the Target Digit on 14% of the trials when the Second-Cued Digit was higher than the Target Digit versus 38% of the trials when the Second-Cued Digit was lower than the Target Digit. Crucially, there was also a main effect of Saliency, B=1.17, SE=.33, Z=3.56, *p* <.001, indicating that participants tended to report the Target Digit if it was more salient than the Second-Cued Digit. Finally, there was Temptation × Saliency interaction, B=.82, SE=.28, Z=2.96, *p* <.01, reflecting that the effect of Saliency was reduced when the Second-Cued Digit was higher than the Target Digit, possibly due to a floor effect (i.e., in that case the number of target reports was low, reducing the effect of Saliency). As in Experiment 1, we found a cluster of “brazen liars” who reported the highest digit, and thus were not sensitive to our manipulations. Removing these participants did not change the results (see Supplementary Material, Fig. 3S) (Fig. 4).

#### Eye movements

Similar to Experiment 1, for seven temporal bins separately, we conducted a GLM with proportion of fixations on the Target Digit as dependent variable, and Temptation, Saliency, and the Temptation × Saliency interaction as fixed effects. We conducted an analogous-but-separate GLM with proportion of fixations on the Second-Cued Digit as dependent variable.

**Fig. 5:**
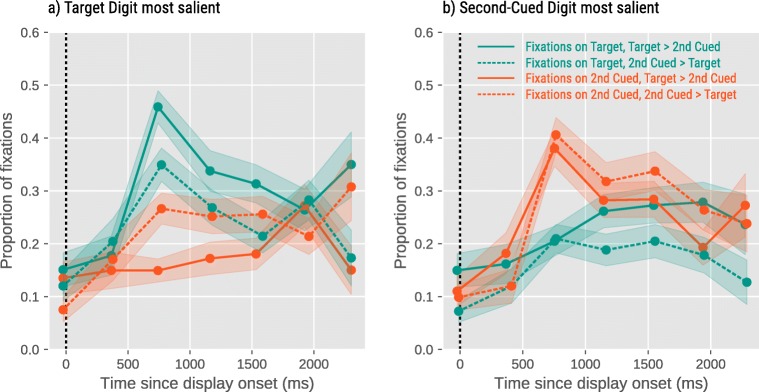
Eye-movement results for Experiment 2. Error bars represent standard errors.

Figure 5 shows that eye movements were driven by the saliency and, although to a lesser extent, the value of the digits. There were main effects of Target Saliency for bin 3, B=.99, SE=.12, Z=8.31, *p* <.0001, and bin 4, B=.37, SE=.14, Z=2.72, *p* <.01, and Temptation for bin 1, B=-.38, SE=.18, Z=-2.10, *p* =.03, and bin 5, B=-.41, SE=.14, Z=-2.86, *p* =.004 on the proportion of fixations on the Target Digit.

Similarly, there were main effects of Target Saliency for bin 3, B=-.83, SE=.12, Z=-6.99, *p* <.0001, bin 4 B=-.49, SE=.14, Z=-3.52, *p* <.001, and bin 5 B=-.48, SE=.14, Z=-3.33, *p* <.001, and Temptation for bin 3, B=.25, SE=.12, Z=2.09, *p* =.04, and bin 4, B=.30, SE=.14, Z=2.14, *p* =.04 on the proportion of fixations on the Second-Cued Digit (see Fig. 5S in the Supplementary Material for the pattern without “brazen liars”).

Thus, by increasing the visual saliency of a stimulus, we were able to guide participants’ gaze and attention toward it, which subsequently increased the probability of participants reporting this digit. The effect of visual saliency on eye movements was especially pronounced around 500–1,000 ms (bin 3) after the onset of the stimulus display. This is relatively slow for an effect that is, by definition, driven by bottom-up stimulus features (e.g., Theeuwes et al., 1998). Presumably, eye movements were slightly delayed in our paradigm because the jittery cue was itself a salient visual event that briefly kept gaze locked to the display center. However, even though with a slight delay, our visual-saliency manipulation had the typical effect of attracting gaze especially strongly early in time, and less so later in time (cf. Donk & Van Zoest, 2008).

## Discussion

When honesty and profit conflict, people often show ethical blind spots; that is, they pay more attention to self-serving (yet immoral) information at the expense of less profitable (yet moral) information (Bazerman & Tenbrunsel, 2011; Pittarello et al., 2015). But when do ethical blind spots arise? And can they be reduced?

In two experiments using eye tracking, we found that – overall – people tend to look quickly at the option that they would later report, even when this option is dishonest (but more profitable). In our paradigm, the first eye movements were made fairly slowly, presumably because attention was first pulled toward the central cue, and it took some time for participants to disengage from this and initiate an eye movement. Therefore, the first eye movements are not reflexive in the same way that, for example, very rapid eye movements toward sudden onsets are reflexive (e.g., Theeuwes et al., 1998). However, the first eye movements likely nevertheless reflect the first step in the attentional selection of the stimuli, and – crucially – this first attentional selection is already biased toward the profitable option. The fact that blind spots arise relatively early in a complex task like ours indicates that our findings would be stronger using simpler cheating tasks where, for instance, participants have to choose between two colors or shapes to determine their pay, or where less attention is allocated to a fixation point preceding choices.

Importantly, we show that it is possible to reduce ethical blind spots by increasing the visual saliency of honest options, thus guiding attention toward them in a bottom-up way (Theeuwes et al., 1998), suggesting that a simple and non-invasive visual intervention can redirect people’s attention and gaze toward morally desirable information.

We contribute to the literature on behavioral ethics in two important ways. First, our study is the first to directly show that people’s attention is quickly biased toward profitable choice options, even if these correspond to dishonest behaviors (see Hochman et al., 2016; Pittarello et al., 2015; converging on the same point). Thus, we extend current work on the attentional processing underlying morality (Fiedler & Glöckner, 2015). Second, we demonstrate a cost-effective intervention to reduce self-serving mistakes. Specifically, we suggest that subtly increasing the saliency of morally preferable options can make people focus more on such information, in turn reducing the tendency to make morally questionable choices.

Some limitations are worth discussing. First, the presence of “brazen liars” (participants who always selected the most profitable option, thus disregarding the task instruction altogether) seems to indicate that some people are not sensitive to either temptation or saliency. While our main findings were not affected by “brazen liars,” their existence may limit the generalizability of our results. Second, it is unclear whether participants noted the saliency manipulation in Experiment 2, and if so, how they reacted to it. Exploring for whom saliency hampers or increases dishonesty would allow for more fine-grained future interventions.

Accordingly, it would be interesting to test different ways to manipulate saliency, and to identify under which circumstances they are more (or less) effective in shaping ethical choices, and whether they could be used to increase accuracy when no incentives to cheat are provided. Finally, a promising avenue would be to test whether psychological traits such as a feeling of entitlement – which recent research found to predict dishonesty (Schurr and Ritov, 2016) – predict the extent to which participants are susceptible to the effect of saliency. Another interesting possibility would be to make externalities (the cost of lying) more salient. It can be argued that participants did not really feel that they were stealing, and thus did not feel guilty for their behavior. One way to address this question would be stressing the fact that rewards would be taken from the experimenters’, or another student’s budget.

In summary, everyday life provides plenty of opportunities for cheating. In such settings, people tend to immediately focus on profitable (yet dishonest) information, showing ethical blind spots. We suggest a potential remedy to such blind spots: Making moral information more salient can reduce convenient “oversights” and foster ethicality.

## Electronic supplementary material

ESM 1 (DOCX 1293 kb)

## References

[CR1] Awh E, Belopolsky AV, Theeuwes J (2012). Top-down versus bottom-up attentional control: a failed theoretical dichotomy. Trends in Cognitive Sciences.

[CR2] Ayal S, Gino F, Barkan R, Ariely D (2015). Three principles to REVISE people’s unethical behavior. Perspectives on Psychological Science.

[CR3] Balcetis E, Dunning D (2006). See what you want to see: motivational influences on visual perception. Journal of Personality and Social Psychology.

[CR4] Bazerman MH (2014). The power of noticing: What the best leaders see.

[CR5] Bazerman MH, Tenbrunsel AE (2011). Blind spots: Why we fail to do what’s right and what to do about it.

[CR6] Chugh D, Bazerman MH, Banaji MR, Moore DA, Cain DM, Loewenstein G, Bazerman MH (2005). Bounded ethicality as a psychological barrier to recognizing conflicts of interest. Conflicts of interest: Challenges and solutions in business, law, medicine, and public policy.

[CR7] Donk M, Zoest W v (2008). Effects of Salience Are Short-Lived. Psychological Science.

[CR8] Evans JSB (2008). Dual-processing accounts of reasoning, judgment, and social cognition. Annual Review of Psychology.

[CR9] Fiedler S, Glöckner A (2015). Attention and moral behavior. Current Opinion in Psychology.

[CR10] Gino F, Schweitzer ME, Mead NL, Ariely D (2011). Unable to resist temptation: How self-control depletion promotes unethical behavior. Organizational Behavior and Human Decision Processes.

[CR11] Greene JD, Paxton JM (2009). Patterns of neural activity associated with honest and dishonest moral decisions. Proceedings of the National Academy of Sciences, USA.

[CR12] Haidt J (2007). The new synthesis in moral psychology. Science.

[CR13] Hochman G, Glöckner A, Fiedler S, Ayal S (2016). “I can see it in your eyes”: Biased Processing and Increased Arousal in Dishonest Responses. Journal of Behavioral Decision Making.

[CR14] Itti L, Koch C, Niebur E (1998). A model of saliency-based visual attention for rapid scene analysis. IEEE Transactions on Pattern Analysis and Machine Intelligence.

[CR15] Jacobsen C, Fosgaard TR, Pascual-Ezama D (2018). Why do we lie? A practical guide to the dishonesty literature. Journal of Economic Surveys.

[CR16] Kahneman D (2011). Thinking, Fast and Slow.

[CR17] Mathôt S, Schreij D, Theeuwes J (2012). OpenSesame: An open-source, graphical experiment builder for the social sciences. Behavioral Research Methods.

[CR18] Mazar N, Amir O, Ariely D (2008). The dishonesty of honest people: A theory of self-concept maintenance. Journal of Marketing Research.

[CR19] Mead N, Baumeister RF, Gino F, Schweitzer M, Ariely D (2009). Too tired to tell the truth: Self-control resource depletion and dishonesty. Journal of Experimental Social Psychology.

[CR20] Orquin, J. L, Scholderer, J., & Jeppesen, H. B (2012). What you see is what you buy: How saliency and surface size of packaging elements affect attention and choice. *SABE 2012*.

[CR21] Pittarello A, Leib M, Gordon-Hecker T, Shalvi S (2015). Justifications shape ethical blind spots. Psychological Science.

[CR22] Pittarello A, Motro D, Rubaltelli E, Pluchino P (2016). The relationship between attention allocation and cheating. Psychonomic Bulletin & Review.

[CR23] Schurr A, Ritov I (2016). Winning a competition predicts dishonest behavior. Proceedings of the National Academy of Sciences.

[CR24] Shalvi S, Dana J, Handgraaf MJ, De Dreu CK (2011). Justified ethicality: Observing desired counterfactuals modifies ethical perceptions and behavior. Organizational Behavior and Human Decision Processes.

[CR25] Shalvi S, Eldar O, Bereby-Meyer Y (2012). Honesty requires time (and lack of justifications). Psychological Science.

[CR26] Shalvi S, Eldar O, Bereby-Meyer Y (2013). Honesty requires time—a reply to Foerster et al. (2013). Frontiers in Psychology.

[CR27] Strack F, Deutsch R (2004). Reflective and impulsive determinants of social behavior. Personality and Social Psychology Review.

[CR28] Theeuwes J, Kramer AF, Hahn S, Irwin DE (1998). Our eyes do not always go where we want them to go: Capture of the eyes by new objects. Psychological Science.

[CR29] Weisel O, Shalvi S (2015). The collaborative roots of corruption. Proceedings of the National Academy of Sciences.

[CR30] Yantis S, Jonides J (1984). Abrupt visual onsets and selective attention: Evidence from visual search. Journal of Experimental Psychology: Human Perception and Performance.

