# Supplementary material for: Visual saliency influences ethical blind spots and (dis)honesty
Source: Psychon Bull Rev. 2019 Jul 6;26(5):1719–28. doi: 10.3758/s13423-019-01638-1 (PMC6797665; doi:10.3758/s13423-019-01638-1)
Supplement: Supplementary file 1 — (DOCX 1293 kb) [file 13423_2019_1638_MOESM1_ESM.docx]

# Visual saliency influences ethical blind spots and (dis)honesty

Andrea Pittarello^1,2^, Marcella Frătescu^2^, and Sebastiaan Mathôt^2^

^1^ Department of Psychology, Brooklyn College, City University of New York

^2^ Department of Psychology, University of Groningen, The Netherlands

**Supplementary Material**

Below, we report additional figures regarding participants’ behavior and eye movements.


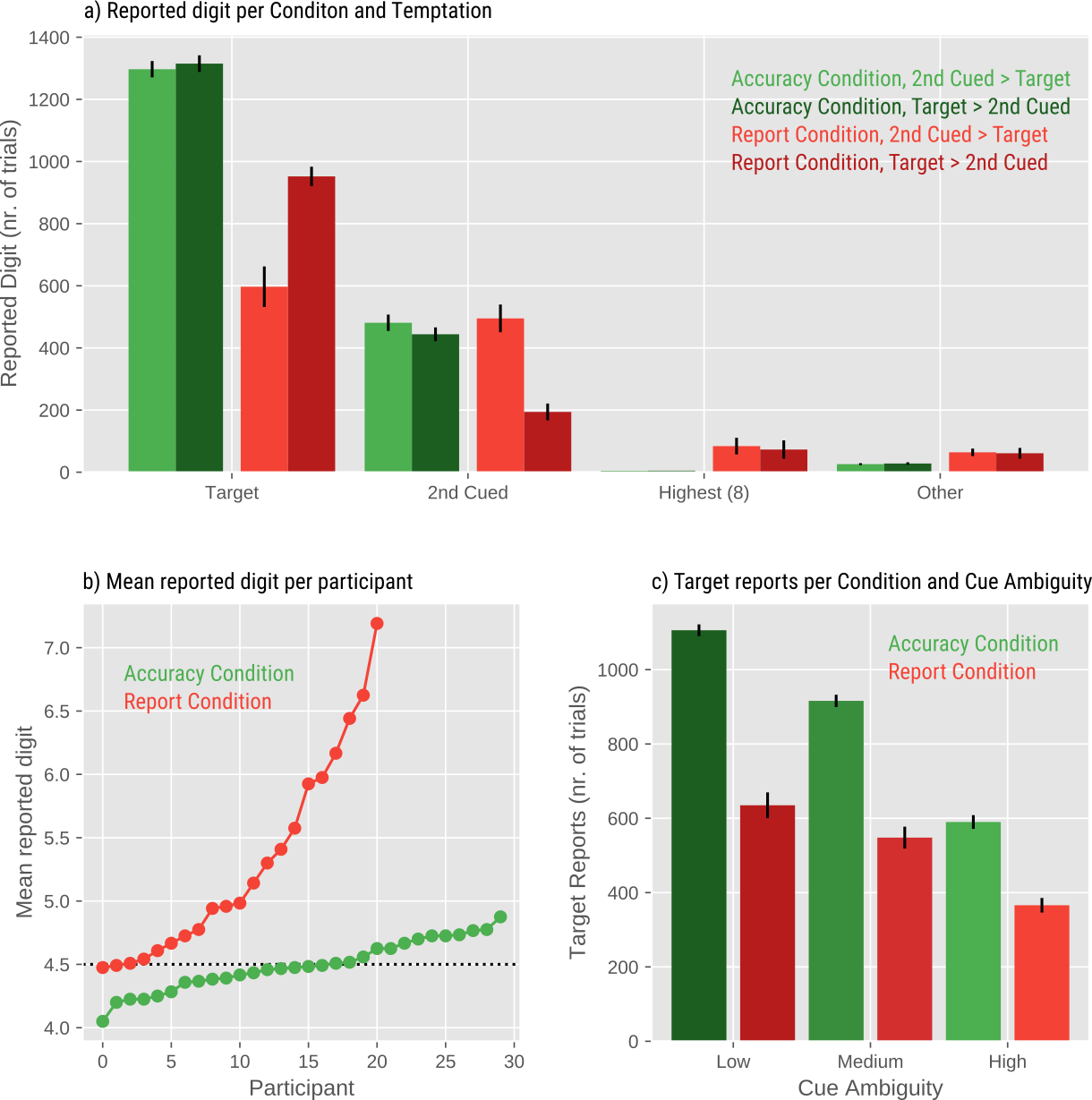


**Figure 1S**: Behavioral results for Experiment 1 after excluding participants whose average reported digit was 7.5 or higher. Error bars represent standard errors.


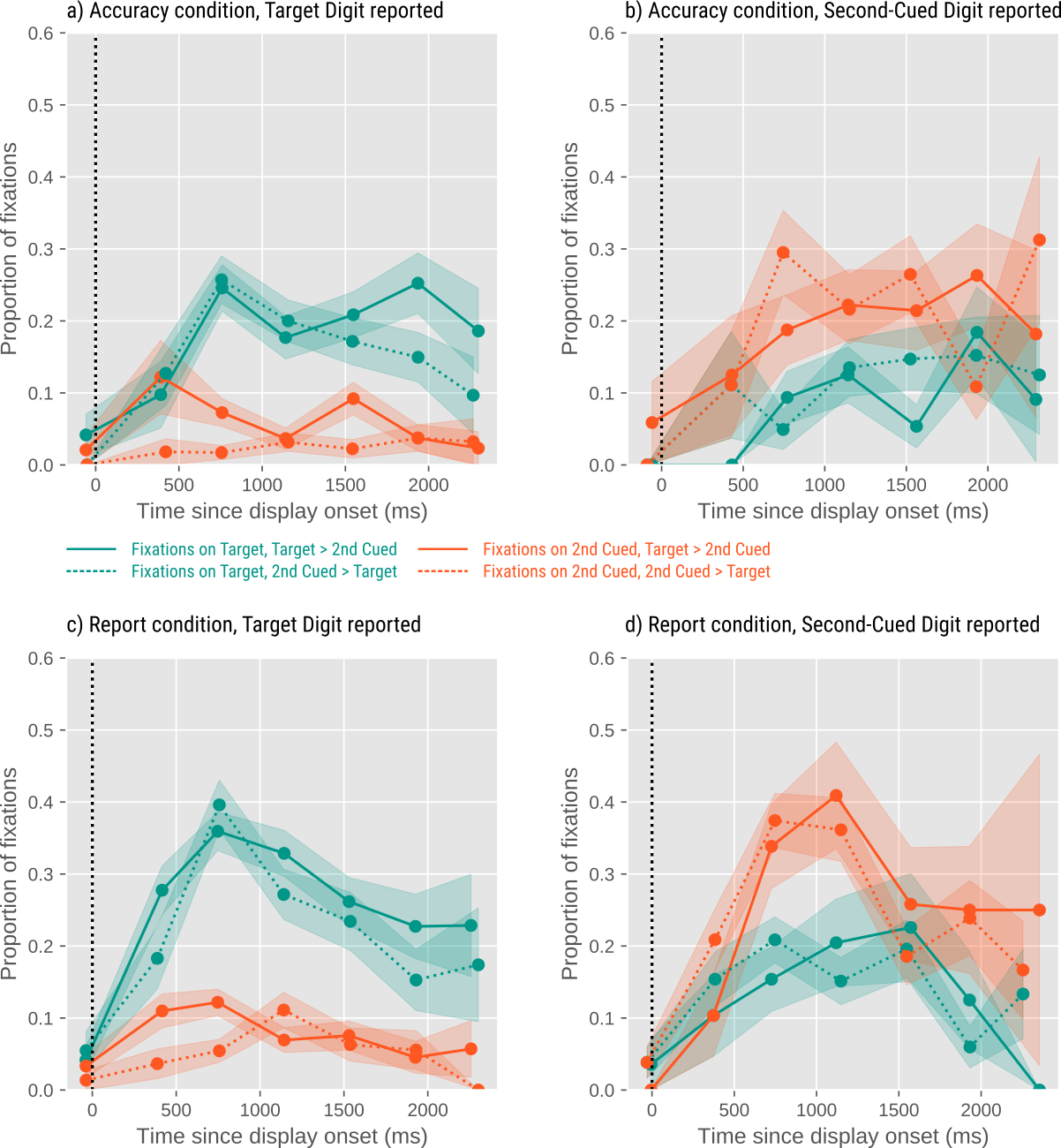


**Figure 2S**. Eye-movement data for Experiment 1 after excluding participants whose average reported digit was 7.5 or higher. Error bars represent standard errors.


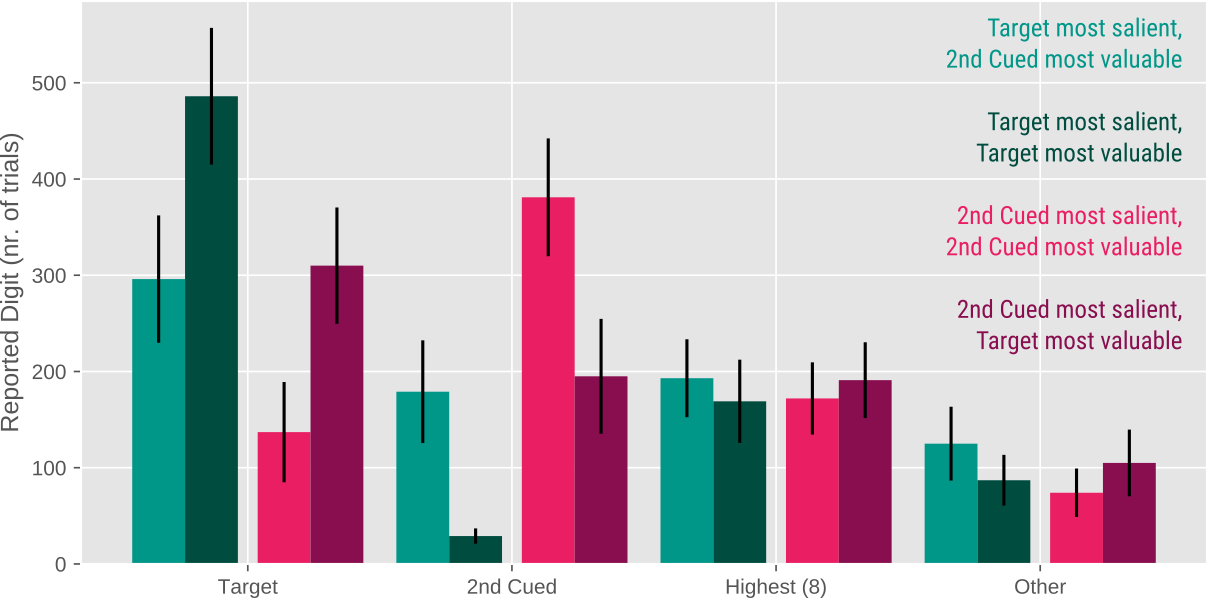


**Figure 3S**. Behavioral results for Experiment 2 after excluding participants whose average reported digit was 7.5 or higher. Error bars represent standard errors.


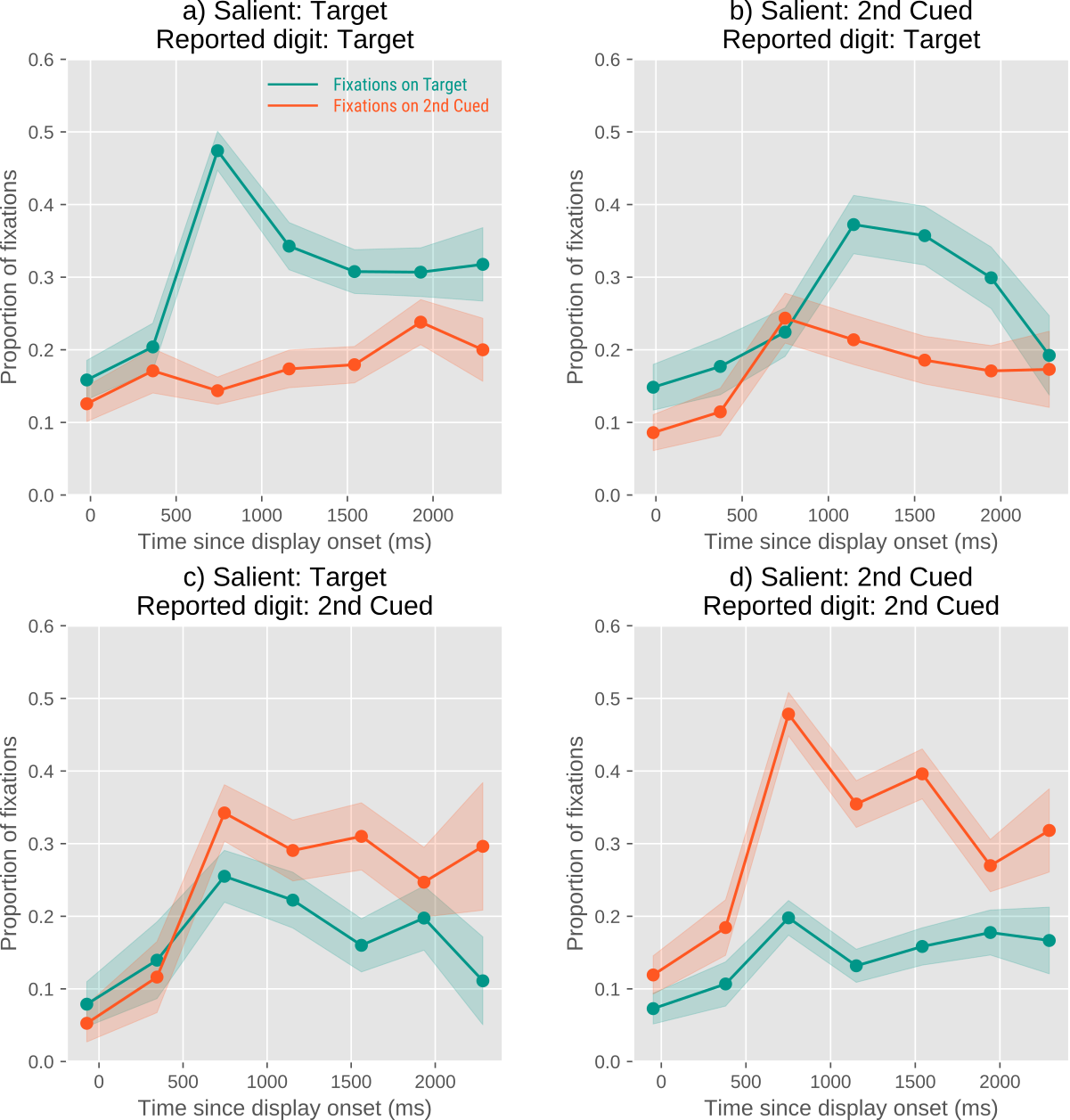


**Figure 4S**. Eye-movement results for Experiment 2 split by reported digit and saliency after excluding participants whose average reported digit was 7.5 or higher. Error bars represent standard errors.


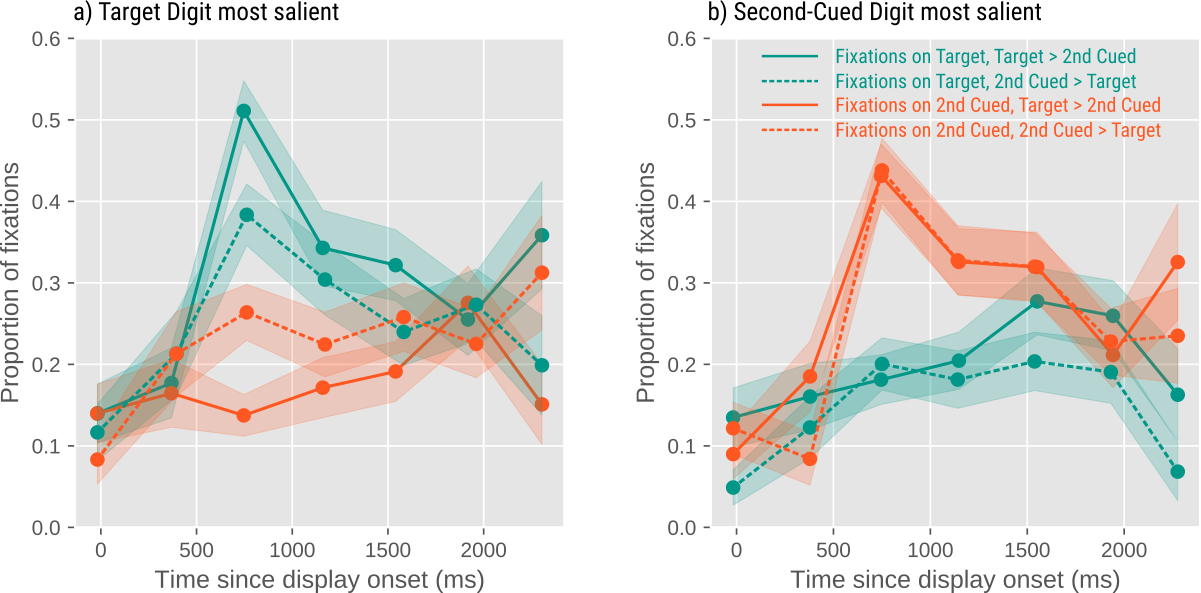


**Figure 5S**. Eye-movement results for Experiment 2 split by saliency and temptation after excluding participants whose average reported digit was 7.5 or higher. Error bars represent standard errors.

**Figure 6S**. Number of Target reports split by condition (accuracy vs. report), ambiguity (low, medium, high) and temptation for Experiment 1 (Note: Second Cued < Target= Second Cued less valuable than Target, Second Cued > Target = Second Cued more valuable than Target.)

**Figure 7S**. Number of Second-Cued reports split by condition (accuracy vs. report), ambiguity (low, medium, high) and temptation for Experiment 1. (Note: Second Cued < Target= Second Cued less valuable than Target, Second Cued > Target = Second Cued more valuable than Target.)


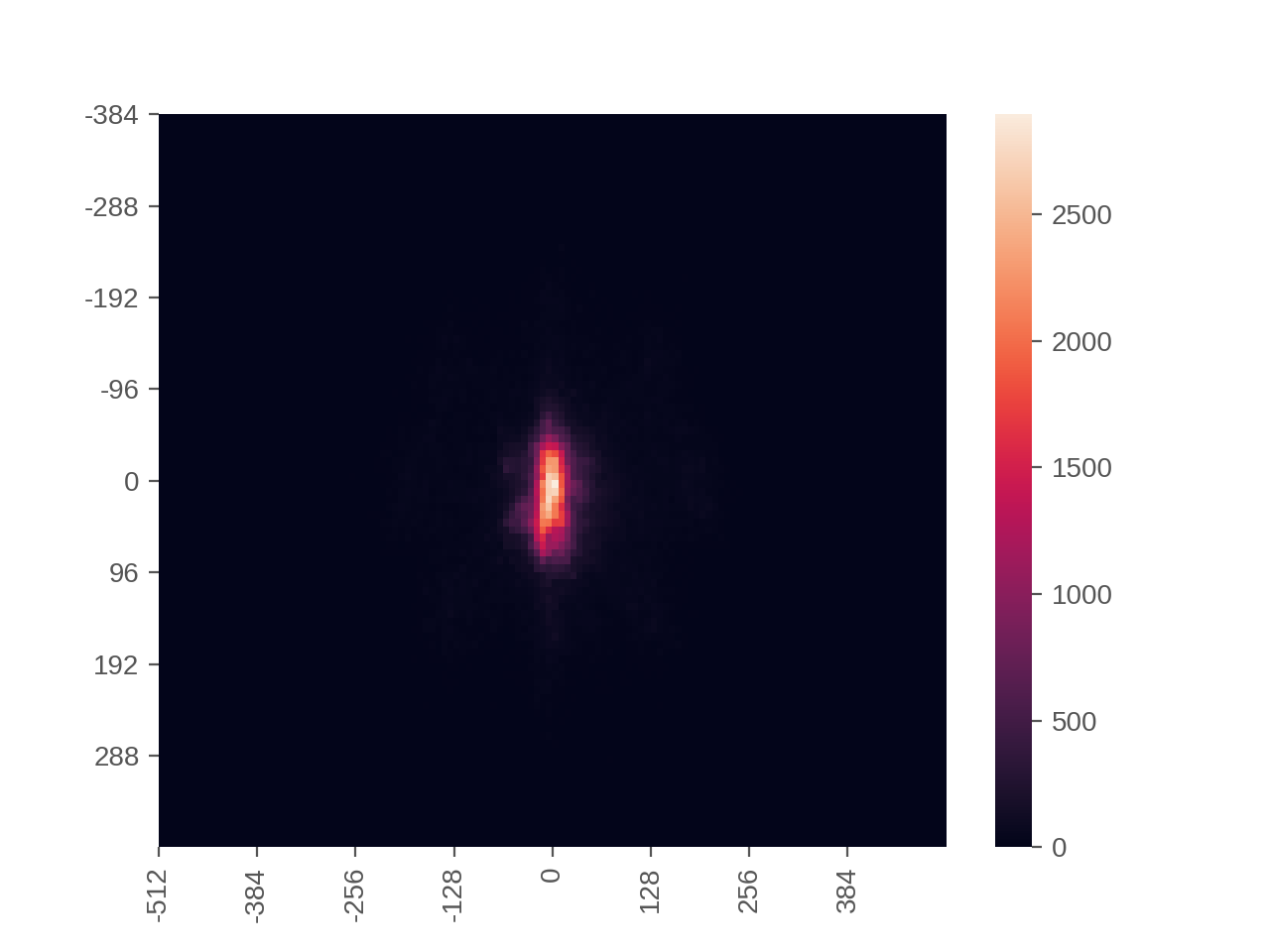


**Figure 8S**. The distribution of gaze position in the 450 ms after the presentation of the target display.
